# The blue tick: WhatsApp as a care tool in pediatric palliative care

**DOI:** 10.1186/s12904-025-01709-2

**Published:** 2025-04-14

**Authors:** Mariangela Rosa, Anna Santini, Valentina De Tommasi, Caterina Agosto, Luca Giacomelli, Simonetta Papa, Lara Vecchi, Franca Benini

**Affiliations:** 1 Pediatric Pain and Palliative Care Service, Department of Women’s and Children’s Health, University Hospital, Padova, Italy; 2 Polistudium SRL, Milan, Italy; 3 Department of Women’s and Children’s Health, University of Padua, Padova, Italy

**Keywords:** Pediatric palliative care, Communication, Instant messaging

## Abstract

**Background:**

Comprehensive care, which includes psychological support for pediatric palliative care (PPC) patients, addresses all aspects of the patient’s life. It focuses on managing disease-related challenges and identifying strategies for personal, relational, and social dynamics. Effective communication between patients and healthcare providers is essential in the PPC setting. While various assistive communication tools have been explored in literature, using instant messaging within PPC remains relatively unexamined. Therefore, this study aims to evaluate the role of this technology in supporting patient care.

**Methods:**

Virtual messages exchanged between psychologists and PPC patients via WhatsApp were analyzed retrospectively. The content of these messages was examined qualitatively through thematic analysis using ATLAS.ti software, and sociodemographic characteristics were also collected.

**Results:**

A total of 25 patients receiving PPC services were recruited, with 5,623 messages evaluated across 766 interaction threads between patients and psychologists. On average, there were 7.34 messages per thread, ranging from 1 to 116 messages per thread. The qualitative thematic analysis revealed that the message content primarily fell into two areas: informative and psychological. The informative area included exchanges about symptomatology, procedures performed, visits, and general health. The psychological area consisted of messages reflecting negative and positive thoughts and emotions related to personal experiences, peer relationships, and family dynamics. Notably, 8.2% of the messages were significant enough to be shared within the team, which helped redirect or modify the care plan for the child and family. Additionally, 8.9% of the messages prompted medical, nursing, or psychological emergency consultations.

**Conclusions:**

This communication tool appears more accessible for adolescent patients who regularly use instant messaging applications. Its immediacy and ease of use make such communication strategies effective and efficient for organizing, coordinating, and implementing care for a sizable portion of PPC patients.

**Graphical Abstract:**

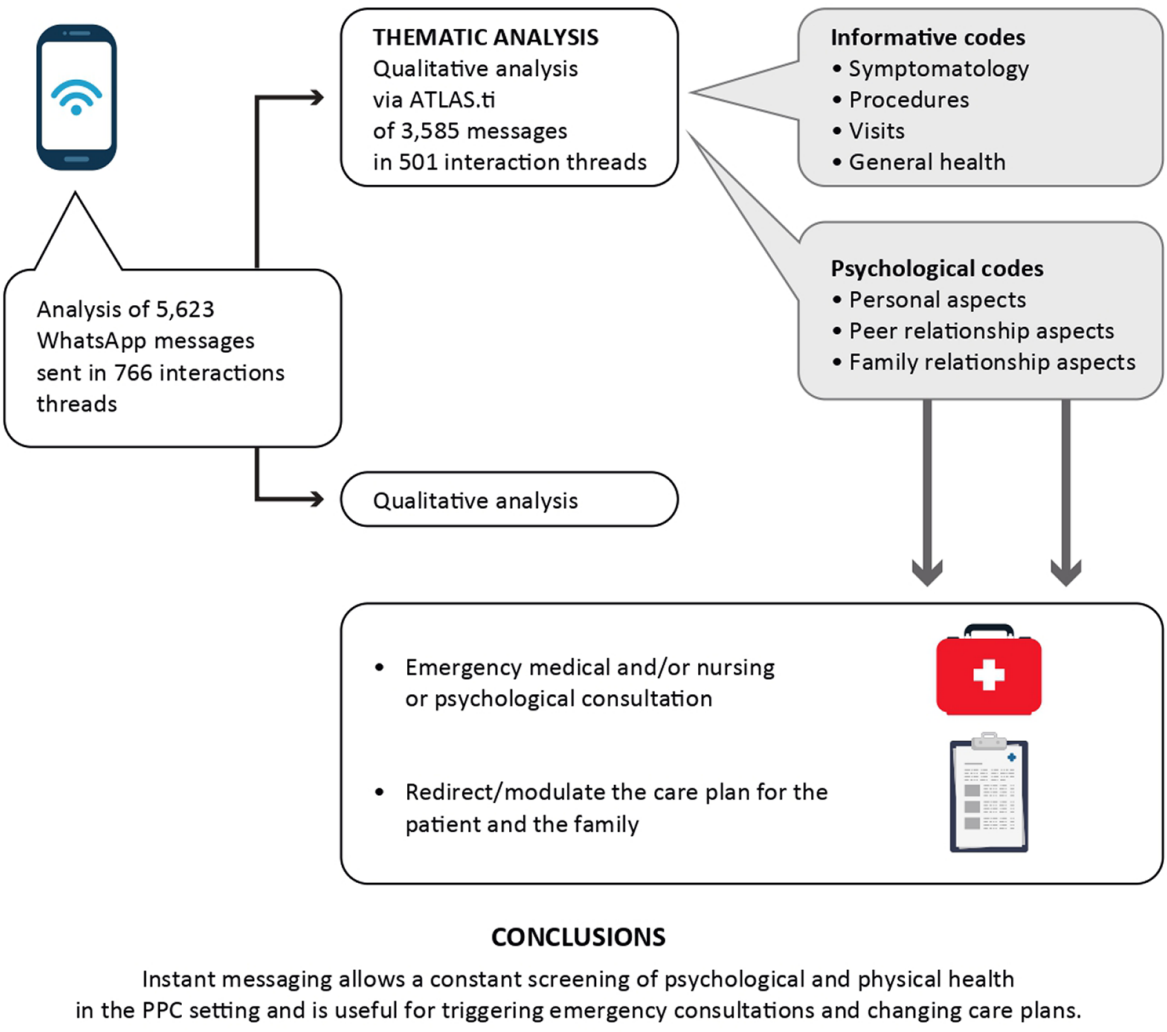

## Background

Pediatric palliative care (PPC) ought to be provided upon the onset of a life-limiting or life-threatening illness, not solely during the end-of-life phase. Palliative care should be maintained for the duration deemed necessary [1].

The clinical and care needs of children in palliative care (PPC) and their families are complex, encompassing physical, psychosocial, and spiritual aspects. Therefore, PPC requires the involvement of a specialized team that addresses each individual’s specific and unique needs [1]. Effective communication between patients and healthcare providers (HCPs) is crucial [2].

Utilizing instant messaging platforms is a well-established communication strategy that enhances patient-centered care and family involvement, ensuring better care coordination [3], even in palliative care [4].

In Italy, the National Council of Psychologists has strongly recommended that professionals deliver their services through digital devices to ensure the continuity of psychological support and treatments, which have demonstrated beneficial effects on patients [5]. Nevertheless, the extended use of these approaches is associated with a significant burden to HCPs [4, 6, 7], and using technology as an assistive communication tool in PPC remains relatively unexplored. Parpia et al. examined the caregiver-professional relationship enabled by digital communication for pediatric patients with complex care needs [8]. However, no research has explored the care connection through instant messaging tools to communicate with their healthcare providers. Additionally, there is no information regarding the burden on healthcare providers using these tools.

The present study analyzed the content of text messages exchanged between psychologists and patients who participated in the PPC network of Padua Hospital (Padua, Italy), a referral center for PPC in northern Italy. Specifically, we focused on the WhatsApp instant messaging application, which we identified as the most widely used platform among young individuals [9, 10] and the healthcare professionals at our institution; whose interchanged and read messages are marked by the blue tick.

The objective was to evaluate the role of instant messaging as a communication, assistance, and care tool for patients in PPC. Additionally, we characterized the type of psychological assistance through qualitative analysis of messages exchanged over a calendar year. This involved quantifying messages and threads exchanged based on criteria such as holidays and weekdays, within and outside the psychologist’s designated working hours, and analyzing interaction times.

## Patients and methods

### Study design

This retrospective review examined both the content and volume of WhatsApp messages exchanged between patients using their smartphones and the referring psychologist of the Padua Pediatric Palliative Care Service from March 31, 2022, to March 31, 2023. We considered a broad period to facilitate collecting and analyzing a substantial number of messages.

The inclusion criteria were as follows: receiving care from the Pediatric Palliative Care Service of Padua, which encompasses oncological diagnoses, non-oncological diagnoses, or undiagnosed cases; being between 10 and 25 years old (with the upper age limit set at 25 since patients may continue in PPC beyond legal adulthood); being proficient in written Italian; and having the ability to use a smartphone independently with the WhatsApp messaging application installed. The exclusion criteria included severe cognitive impairment, inability to comprehend and write in Italian independently, and a lack of technological proficiency to use a smartphone without parental assistance.

### Data collection and variables considered in the analysis

The messages deemed relevant to the therapeutic relationship were analyzed qualitatively and through descriptive analysis. In contrast, the other messages were categorized as “Miscellaneous” and included solely in the quantitative thread analysis. Furthermore, the socio-demographic characteristics of the participants were collected and examined.

Sociodemographic data were stratified by age, sex, and disease. Disease type was also categorized into oncological, non-oncological, and undiagnosed condition macro-categories [1].

Qualitative data analysis was carried out rigorously and systematically using the software ATLAS.ti [11] which is widely used in medical and health research [12]. Following the principles of grounded theory [13–15], two authors (MR and AS) classified each text into themes. The research team reviewed coding to identify similarities and differences between MR and AS. Each new piece of information collected was compared with prior data, allowing us to refine categories and their interrelationships. The conceptual categories that emerged from the analyses reflect the key themes in the data. These categories were developed gradually as new concepts (codes) emerged from the data. Data collection continued until theoretical saturation was achieved, meaning no significant new codes surfaced [16, 17]. ATLAS.ti assisted in organizing the data, analyzing the relationships between categories, and visualizing the analysis results.

In qualitative terms, WhatsApp messages and interaction threads (which indicate a potential series of discussion messages between the patient and the psychologist on a specific topic) were analyzed based on the conceptual categories identified by ATLAS.ti.

Additionally, messages and threads that significantly redirected or modified the treatment plan for the youth and their family were tracked. Examples of these messages include those concerning authorization for an electric wheelchair and sharing information about adding a new supplement to their therapeutic plan. The analysis also considered messages that prompted emergency consultations, whether globally or related explicitly to psychological matters. Examples of these messages include those that activate a psychological consultation to initiate local psychological support or to discuss urgent family issues affecting the patient, a patient’s request to transition from a tracheostomy to a mask that should be reported promptly to the doctor, and an increase in pain requiring adjustments to analgesic therapy that need to be communicated immediately to the doctor to ensure the patient’s well-being. The time and date of each message were categorized into weekdays (Monday to Friday) or holidays (Saturday, Sunday, national holidays), and messages sent or received “within” (9:00 AM to 5:00 PM) or “outside” (before 9:00 AM and after 5:00 PM) the working hours of the referring psychologist. Finally, the “interaction time” refers to the total hours and average time spent reading and managing messages during the 12-month interaction period of this study.

### Descriptive statistical analysis

Continuous variables are presented as medians or means, while categorical variables are shown as counts and percentages.

## Results

### Socio-demographic characteristics of the sample

As of March 2023, the PPC network had 258 patients, 63 of whom interacted directly with psychologists through instant messaging. However, only 25 of these patients met the inclusion criteria.

Although the inclusion criteria covered individuals aged 10 to 12, we found that youths in this age range preferred engaging through alternative communication methods (e.g., phone calls) rather than instant messaging. Additionally, their primary caregivers often facilitated WhatsApp interactions with these patients, which became an exclusion criterion. Therefore, the instant messaging analysis focused on youths aged 13 and older.

The mean age of the recruited patients was 16.2 years (SD = 2.7; range 13‒24), with a higher frequency of individuals aged 13‒18 years (*n* = 22/26; 84.62%). There was a slightly higher percentage of females (*n* = 13/25; 52.0%), and 92% of the patients under care had non-oncological diseases (rare genetic diseases, muscular disorders, neurological conditions), while 4% had oncological diseases, and 4% had an “undetermined diagnosis” (Table 1).

**Table 1:** Demographic characteristics of the patient population

Characteristics of patients		*n* (%)
Age (years), mean ± SD (median)		16.2 **±** 2.7 (15.0)
Age Groups	13‒14 years	7 (28.0%)
	15‒18 years	15 (60.0%)
	> 18 years	3 (12.0%)
	Total	**25** (**100.0%**)
Sex	Male	12 (48.0%)
	Female	13 (52.0%)
	Total	**25** (**100.0%**)
Patient diagnosis	Oncological	1 ()
	Non-oncological	23 (92.0%)
	Without specific diagnosis	1 (4.0%)
	Total	25 (100.0%)

### Message and thread analysis

From March 31, 2022, to March 31, 2023, a total of 5,623 messages were exchanged across 766 interaction threads between patients and psychologists, averaging 7.34 messages per thread (SD = 8.53), with a range of 1 to 116 messages per thread. In 31 instances, some patients sent a single message to their psychologist at night or on holidays when the practitioner’s phone could not guarantee an immediate response. The patient’s interlocutor replied to these messages on the next workday during business hours, but they were still counted within a “potential.” thread.

In total, 3,585 messages from 501 interaction threads were considered relevant to the therapeutic relationship and were analyzed qualitatively. In contrast, the remaining 2,038 messages from 265 threads were classified as “Miscellaneous.”

### Thematic analysis: message contents

**Fig. 1:**
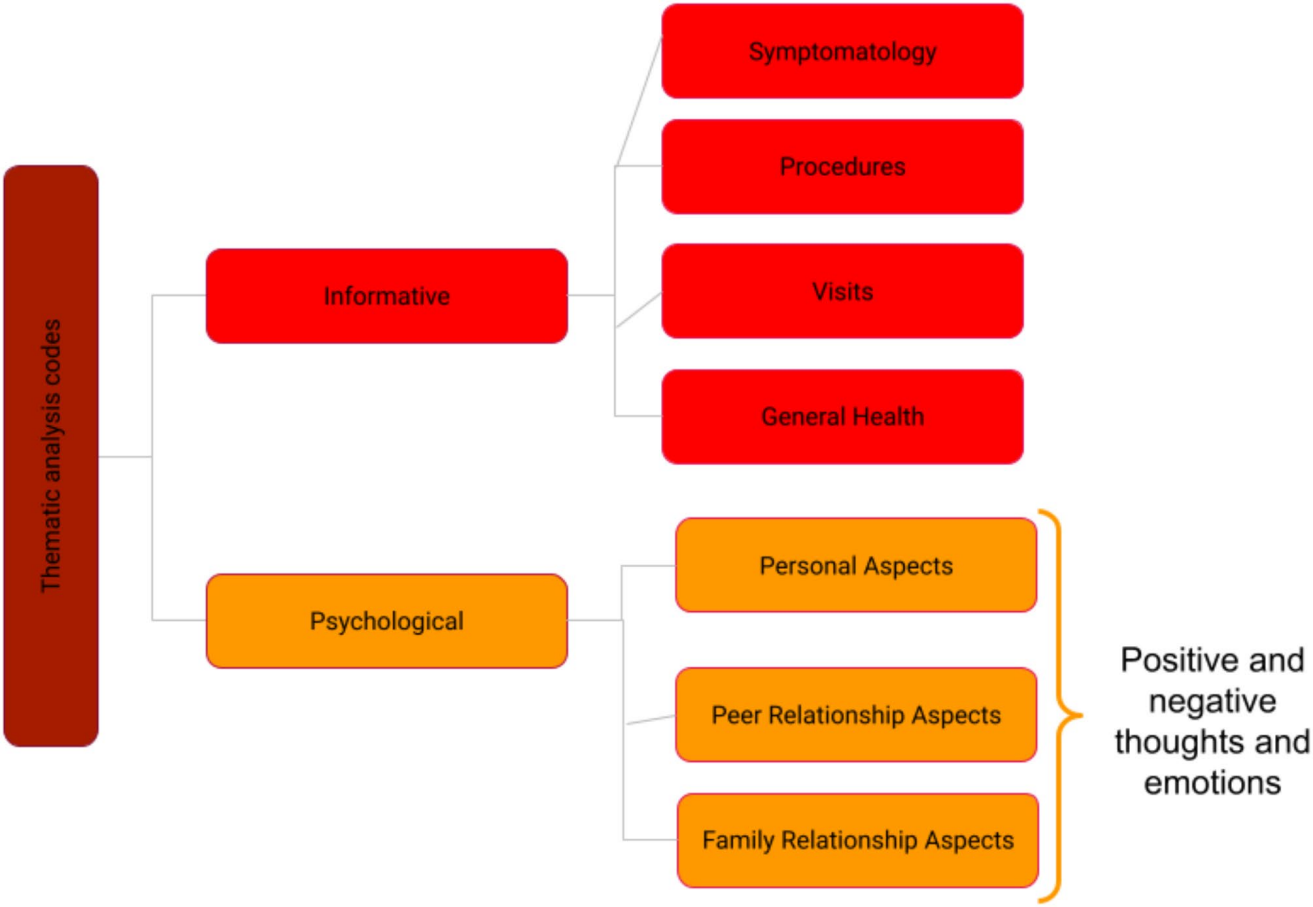
Codes emerged from thematic analysis

The content of messages exchanged between patients and psychologists was analyzed qualitatively using ATLAS.ti (Scientific Software Development GmbH). Figure 1 illustrates the codes that emerged from the analysis.

Communicative exchanges related to the four codes—“Symptomatology,” “Procedures,” “Visits,” and “General Health”—were categorized under the “Informative” code, as they mainly offered guidance for monitoring and clinical assistance coordination.

An example of a message coded as symptomatology was “All these medicines make me very sleepy and hurt my stomach…”; “I have been in the hospital in Treviso these days for a respiratory crisis…”.

The researchers categorized the remaining three codes—“Personal Aspects,” “Peer Relationship Aspects,” and “Family Relationship Aspects”—as part of the “Psychological” category. In the “Peer Relationship Aspects,” we found that the focus was on the emotions and thoughts that arise during interactions with friends and classmates and the challenges of sharing one’s experiences with peers. The “Family Relationship Aspects” explores emotions and thoughts related to relationships with parents, siblings, or other family figures.

This classification includes narratives reflecting personal experiences, explicitly addressing self-perception, self-esteem, and the challenges youth face in managing emotions and daily situations. It emphasizes the youth’s internal experiences, including nightmares and somatization.

An example of a message coded as personal aspects was: “Today I had nightmares…can I tell you about them?“.

**Fig. 2:**
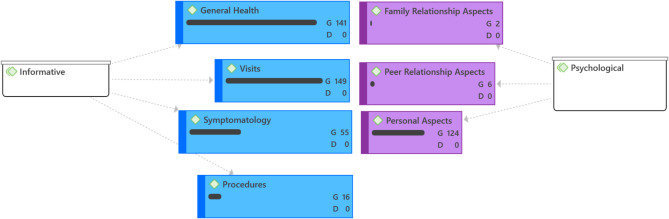
Output of code frequency in care communication between psychologist and patient (D = “density”; G = “groundedness”)

The first output (Fig. 2) illustrates the distribution of the seven codes and the frequency with which message exchange scripts emerged from the thematic analysis in ATLAS.ti. The “groundedness” of a category code was determined by the cumulative number of quotations encoded by all subcodes. It’s important to note that the total count for a category may differ, as multiple codes can be applied to the exact quotation, resulting in a total count that does not simply reflect the sum of the quotations for all subcodes.

Code density is not dependent on the code hierarchy or the number of codes. It is the number of connections between two codes.

**Fig. 3:**
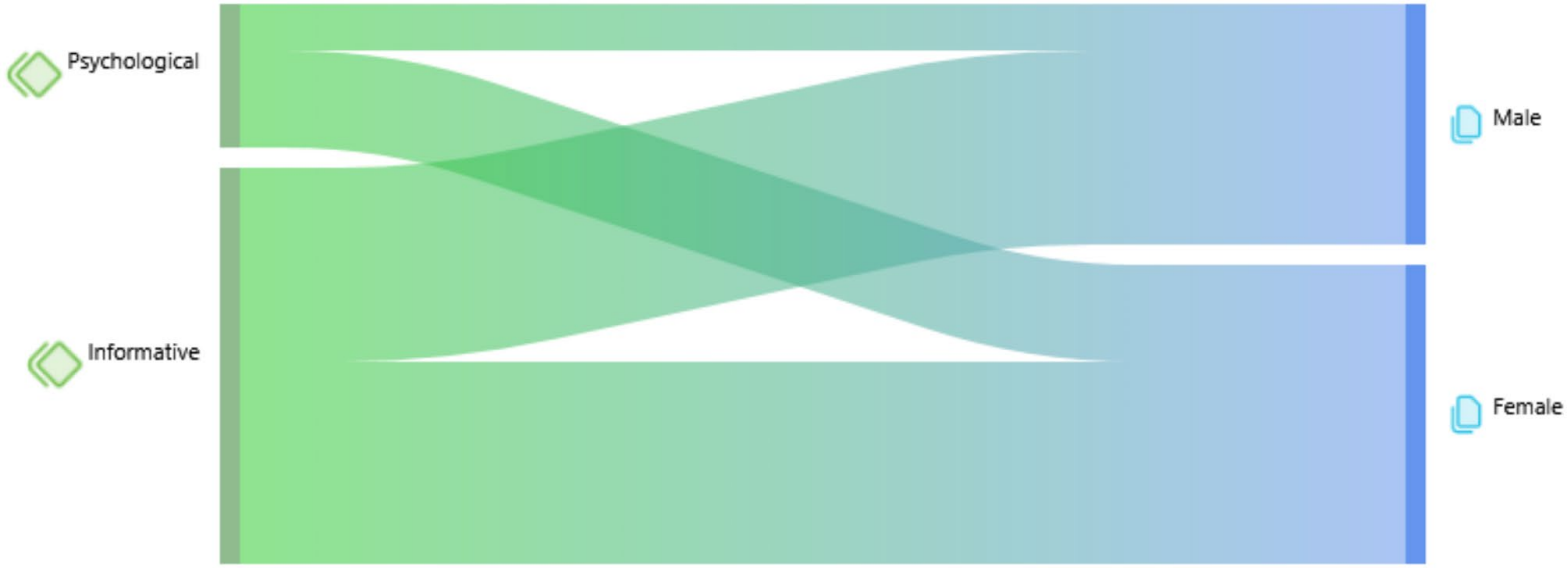
Output of code category co-occurrence and sex

The second output (Fig. 3) indicates that both groups of youths (males and females) engaged more with their designated psychologists on topics related to the “Informative” code category than in communicative interactions associated with the “Psychological” code category. There is a higher co-occurrence of codes connected to the “Psychological” code category in the interaction message scripts of females compared to males. Conversely, the co-occurrence between sex and codes related to the “Informative” code category seems equivalent. Table 2 defines each message type corresponding to each code and provides an example of the message type.

**Table 2:** Type of messages exchanged

Code Message	Definition of Message Type	Example of Message Type
**Informative**		
Symptomatology	Information on physical symptoms, well-being, or discomfort related to aspects of the pathology or associated aids	*“I always have burning and cramping in my stomach*,* and yesterday*,* I had diarrhea right after eating and started sweating. The other night*,* I was in bed*,* started sweating*,* and felt like fainting again because of stomach cramps.”*
		*“I have a lot of discomfort from the new mattress after surgery.”*
Procedures	Information and/or organization related to procedures undergone or procedures/visits to other departments or hospitals in the regional/national territory	*“Hello*,* I am going to Treviso*,* they called us for a video fluoroscopy…"*
Visits	Organization and outcomes of medical visits (day hospital Hospice admission)	*“Good morning*,* doctor*,* do you remember me? I am A.* *I would love to have a chat with you*,* when can we talk?”*
	Scheduling meetings/appointments (in-person/remote) with a psychologist; home visits (psychologist, psychologist + doctor)	
General Health	Information about general well-being	*“Anyway*,* I am doing fine… I measured my oxygen saturation*,* and it is a bit low.”*
**Psychological**		
Personal Aspects	Positive or negative emotions and/or thoughts regarding school, daily life, returning home after procedures/hospitalization, nightmares, and/or somatization related to operations/interventions	*“…I know that tomorrow I will cry a little because my life sucks; when I go to school*,* they tell me to die*,* so it seems like everyone wants me dead; I know my life sucks*,* and it does not make sense. I have many problems*,* they have nothing. It is better to have a shitty life*,* and tomorrow I know everyone will make fun of me…"*
	Exploration/sharing of symptoms and concerns → managing uncertainty	
Peer relationship aspects	Emotions and/or positive or negative thoughts regarding sharing personal/disease aspects with peers	*“… Let’s say I have this discomfort talking to other boys or girls in the hospital*,* I do not know why… I start having not-so-positive feelings as if I want to run away from that person and never talk to them again*,* even though they just want to make friends and be nice to me.”*
Family relationship aspects	Emotions and/or positive or negative thoughts regarding intrafamily relationships	*“Better this way… so my dad and I do not fight because it is always like this lately.”*

### Descriptive statistical analysis of messages

Out of a total of 3,585 messages transmitted, 67.5% are categorized as “Informative,” whereas 32.5% are designated as “Psychological.” The code with the highest frequency of messages among those analyzed is “Visits” (30.6%), followed closely by “Personal Aspects” (31.2%); the former is classified under the “Informative” category, while the latter falls under the “Psychological” category. In summary, most of the messages coded through qualitative analysis were initiated by patients (*n* = 1,974/3,585; 55.1%) to their psychologist. The frequencies and percentages of all the codes examined are detailed in Table 3.

**Table 3:** Descriptive statistical analysis of messages exchanged by thematic codes

Code category	Thematic codes	Patient, *n* (%)	Psychologist, *n* (%)	Total, *n* (%)
Informative, 2,421 (67.5%)	Symptomatology	313 (8.7%)	197 (5.5%)	510 (14.2%)
	Visits	571 (15.9%)	527 (14.7%)	1,098 (30.6%)
	Procedures	33 (0.9%)	33 (0.9%)	66 (1.8%)
	General Health	390 (10.9%)	357 (10.0%)	747 (20.8%)
Psychological, 1164 (32.5%)	Personal Aspects	644 (18.0%)	473 (13.2%)	1,117 (31.2%)
	Peer Relationship Aspects	16 (0.45%)	16 (0.45%)	32 (0.89%)
	Family Relationship Aspects	7 (0.2%)	8 (0.2%)	15 (0.4%)
Total		1,974 (55.1%)	1,611 (44.90%)	3,585 (100.0%)

Furthermore, it was noted that the quantity and category of messages pertinent to the therapeutic relationship differed according to the sender’s gender: girls transmitted a more significant number of messages (*n* = 1,254/1,974; 63.5%) in comparison to boys (*n* = 720/1,974; 36.5%). Notably, girls communicated more frequently with the psychologist regarding topics classified under the “Personal Aspects” code (*n* = 519/1,254; 41.4%) than boys, who demonstrated a higher propensity to send messages associated with the “Symptomatology” code (*n* = 225/720; 31.3%). Both genders predominantly interacted with the psychologist within the “Visits” domain, with boys sending 197 out of 720 messages (27.4% of total messages) and girls sending 374 out of 1,254 messages (29.8% of total messages).

In thirty-six interaction threads, 8.9% of the messages exchanged between patients and psychologists (*n* = 319 out of 3,585) necessitated medical or psychological emergency consultation. Furthermore, 8.2% of the messages (*n* = 293 out of 3,585) across twenty-eight interaction threads were referred by the psychologist to the team, facilitating a collegial discussion to redirect or modulate the care plan for the patient and their family. Within five interaction threads, 1.1% (*n* = 41/3585) of the messages activated an emergency medical/nursing consultation and a psychological consultation while simultaneously redirecting the psychologist to the team. This facilitated a collegial discussion aimed at redirecting and modulating the treatment plan for the patient and their family.

### Evaluation of message threads and interaction time

The aggregate interaction duration between the patient and psychologist throughout the examination, encompassing 776 exchanges, amounted to approximately 103 h and 52 min. This comprised nearly 98 h of interaction during weekdays and approximately 5 h during weekends and holidays. Additionally, on average, each interaction transpired in roughly one minute (approximately 1 min and 1 s). This latter statistic illustrates the promptness with which the psychologist addresses and responds to the immediate needs articulated by the patient via WhatsApp. Nevertheless, it is imperative to underscore that the interaction duration becomes significant within the context of the therapeutic engagement year.

The average interaction time for each area in a year was approximately 12 h and 59 min. The majority of patient-psychologist interaction threads (723/766, 94.4%) were sent on workdays (Monday to Friday), of which 9.8% (*n* = 71/723) were sent outside working hours (Table 4).

**Table 4:** Descriptive and average time per interaction area on workday/holiday and within/outside working hours

		*n*	%	Average time (hours) per interaction area
Weekday, 723 (94.4%)	Within	652	90.2	88.42
	Outside	71	9.8	9.63
Holidays, 43 (5.6%)	Within	0	0.0%	0
	Outside	43	100.0%	5.83

Notably, there is an average of 16 h of potential work outside the psychologist’s regular hours, either on weekends or holidays, averaging nearly 6 h (5.83 h), or during weekdays but beyond the psychologist’s working hours, averaging almost 10 h (9.63 h). The psychologist faces a significant workload extending beyond regular workdays into off-hours.

We discovered that slightly over half (*n* = 38/710; 53.5%) of the interaction threads on workdays, outside the psychologist’s working hours, consisted solely of patient messages without any response from the psychologist until the end of the day. This situation led to an increased workload for the practitioner the next day. Additionally, only 41.9% (*n* = 18/43) of interaction threads over holidays and weekends featured at least one response from the psychologist, leaving the majority (58.1%; *n* = 25/43) unanswered. The psychologist typically addressed these requests in the following week (Table 5).

**Table 5:** Descriptive threads with no response or at least one response from the psychologist “outside” working hours on a workday/holiday

Psychologist replies = 0	*n*	%	Psychologist replies ≠ 0	*n*	%
No. interactions Psychologist = 0	38	53.5	No. interactions Psychologist **≠** 0	33	46.5
“Outside working hours on workday”			”Outside working hours on workday”		
No. interactions Psychologist = 0	25	58.1	No. interactions Psychologist **≠** 0	18	41.9
“Outside on holidays”			“Outside on holidays”		

## Discussion

The use of technology-based communication methods has been minimally explored in pediatric settings [8, 18, 19], involving primary caregivers [8] or directly engaging pediatric patients [18, 19] in interaction with one or more specialized HCPs for their condition.

Due to the insufficient knowledge in this area, this study examines the utilization of instant messaging applications as a method of care for youths receiving palliative psychological care (PPC) and their appointed psychologists. Our research indicates that instant messaging applications facilitate direct interaction between patients and psychological professionals, thereby underscoring the continuity of the care process. The immediacy of communication not only simplifies but also accelerates remote care correspondence for young individuals, optimizing the methodologies employed by psychologists and enhancing the patient’s perception of their active involvement in the therapeutic relationship. Consequently, instant messaging is a significant instrument for communication, support, and care for youths undergoing PPC treatment.

Comprehensive psychological and global assistance to patients adapts to all aspects of the patient’s life, aiding in addressing various conversation topics, as suggested by the message content analysis. Individuals not only focus on aspects concerning their physical health and related practices (visits, procedures) but also share information regarding potential personal and/or relational difficulties (with peers, within the family) resulting from the disease, seeking discussion of strategies and emotional support. Consequently, instant messaging may assist in the management of interpersonal relationships and emotional expressions, including performance anxiety, concerns regarding social interactions with peers, as well as the impacts of agitation or anxiety experienced concerning intrusive phenomena such as nightmares that emerge during communication between the patient and the psychologist. Furthermore, instant messaging can help the child share thoughts, emotions, memories, and wishes, thus promoting their psychosocial well-being [20]. The extensive use of messaging for clinical coordination and organization is fascinating, as evidenced by the substantial volume of messages (*n* = 5,668) and threads (*n* = 773).

In numerous instances, these communications elicited direct clinical responses from healthcare team members, including emergency medical or nursing consultations, psychological consultations, and collegial discussions to refine or adapt the patient and family care plan. This outcome suggests that instant messaging possesses significant practical implications within the healthcare sector. Furthermore, it is noteworthy that the cumulative interaction time between patients and psychologists during the examined year amounted to approximately 103 h and 52 min. On average, each interaction lasted around one minute and thirty seconds (approximately one minute and one second). Lastly, the temporal analysis revealed that the psychologists’ responsibilities extend beyond standard working hours, as active interactions between psychologists and adolescents occur during weekdays and holidays. In summary, a retrospective examination of the interactive exchanges between adolescents and psychologists highlights the significant advantages of these engagements.

Moreover, our findings are consistent with the observations made by Parpia et al. in 2023 [8] regarding secure messaging between caregivers of youth with medical complexities and the healthcare team, which has enhanced their collaborative relationship and empowered parents in their caregiving responsibilities.

## Limitations

Several limitations characterize this retrospective study. The primary limitation pertains to the small sample size (*n* = 25) of eligible participants evaluated in the study. The exclusion criteria, which prioritize the autonomy of youth in engaging with the psychologist via the WhatsApp instant messaging application, further diminished the initial pool of potential participants (*n* = 63), which was already considered relatively minor. A secondary limitation pertains to using WhatsApp, a wireless technology that facilitates communication via end-to-end encryption, thus upholding user data confidentiality. However, it offers diminished privacy compared to secure messaging platforms founded on robust security and privacy protocols, such as the Health Insurance Portability and Accountability Act (HIPAA) [21].

Additionally, WhatsApp enables exclusive message exchanges with a specific recipient without interaction, unlike messaging platforms with more healthcare reference figures [18]. Nonetheless, WhatsApp may be regarded as a more advantageous tool due to its straightforwardness, flexibility, and intuitiveness compared to a messaging platform that necessitates access via a web portal through credentials, which may sometimes require additional instructions for practical usage and access. Furthermore, the WhatsApp application is predominantly downloaded and utilized by adolescents and young adults on their smartphones rather than through a web platform. It includes access and a graphical interface that might be more conveniently coded via a personal computer.

## Conclusions and future prospectives

This study indicates that instant messaging fosters direct and active participation among young individuals in conjunction with healthcare providers, facilitating their articulation of needs and thoughts. The findings present new research avenues concerning using digital technologies within care pathways. Specifically, this study aims to stimulate interest in these communication methods, which can influence the care pathway and are frequently undervalued or underutilized due to their complexity management.

Future research should focus on integrating and enhancing these technologies, examining how they can continue to impact health and well-being. This should involve specific parameters that better assess the tool’s efficiency in light of potential response delays or when using other communication methods, such as phone calls. Additionally, investigating the communication dynamics between various health professionals and caregivers would be valuable; caregivers often utilize instant communication to share information and support their children while interacting with the professionals involved.

**Table:** 

Box 1: Summary of the benefits of using instant messaging in the PPC context
• It enables constant and active screening of symptoms and general health.
• It promotes active participation in organizing visits and procedures.
• It allows the identification of requests for emotional and psychosocial support to manage both negative and positive emotions and thoughts related to personal, social, and family life aspects.
• It allows the child to share thoughts, emotions, memories, and wishes.
• It can trigger requests for emergency consultations for the redirection/modulation of the care plan (e.g., change in the medication dosage, initiation of medical device use, or switching to a different medical device).

## Data Availability

No datasets were generated or analysed during the current study.

## Abbreviations

PPC: Pediatric palliative care
HCP: Healthcare provider
